# The Treatment Decision-making Preferences of Patients with Prostate Cancer Should Be Recorded in Research and Clinical Routine: a Pooled Analysis of Four Survey Studies with 7169 Patients

**DOI:** 10.1007/s13187-020-01867-2

**Published:** 2020-09-17

**Authors:** Andreas Ihrig, I. Maatouk, H. C. Friederich, M. Baunacke, C. Groeben, R. Koch, C. Thomas, J. Huber

**Affiliations:** 1 Division of Psychooncology, Department of General Internal Medicine and Psychosomatic, University Hospital of Heidelberg, INF 410, 69120 Heidelberg, Germany; 2 Department of Urology, Medical Faculty Carl Gustav Carus, TU Dresden, Dresden, Germany

**Keywords:** Treatment decision-making, Prostate cancer, Health services research

## Abstract

Different patients want to take different roles in the treatment decision-making process; these roles can be classified as passive, collaborative, and active. The aim of this study was to investigate the correlation between decision-making preferences among patients with prostate cancer and personal, disease-related, and structural factors. In four survey studies, we asked 7169 prostate cancer patients about their decision-making preferences using the Control Preferences Scale (CPS) and collected clinical, psychological, and quality-of-life measures. Most patients (62.2%) preferred collaborative decision-making, while 2322 (32.4%) preferred an active role, and only 391 (5.5%) preferred a passive role. Age (*p* < 0.001), data collection mode (*p* < 0.001), peer-to-peer support (*p* = 0.018), treatment status (*p* < 0.001), performed or planned radical prostatectomy (*p* < 0.001), metastatic disease (*p* = 0.001), and quality of life (*p* < 0.001) showed significant associations with patients’ preferred decision-making roles. Oncologic risk group, anxiety, and depression were not significant in the model. In particular, younger prostate cancer patients with higher quality of life completing an online survey want to play a more active role in treatment decision-making. Before treatment has started, patients tend to prefer collaborative decision-making. Few prostate cancer patients in Germany prefer a passive role. These patients are mostly older patients, patients with a metastatic disease, and patients who have opted for prostatectomy. Whether this finding reflects a generational effect or a tendency by age group and disease phase should be investigated. Further research is also needed to describe the causalities of these relationships. The CPS offers valuable information for personal counselling and should be applied in clinical routine. In a large group of patients with prostate cancer, we found that there is a strong desire for joint decision-making with the physician before the actual treatment. Especially younger men, men with active online behaviour, and men with a high quality of life want to be actively involved in therapy decision-making processes.

## Introduction

Adequate involvement of patients in treatment decision-making is an important goal of personalized medical care to offer each patient his or her desired role in the treatment course. Patients want to take different roles in the treatment decision-making process; these roles can be classified as passive, collaborative, and active. The Control Preferences Scale (CPS) offers a simple and fast way to assess such individual preferences [1], but it is rarely used in clinical routine. There is some evidence from research studies showing that an active role is associated with better values on patient-reported outcome measures [2–5].

Two large population-based studies deal with decision-making preferences [6, 7]. In a German cohort study, 3312 adults older than 64 years were recruited by their general practitioners in the course of a regular health check-up. Asked for their individual treatment decision-making preferences, 46% preferred an active role, 30% preferred a collaborative role, and 24% preferred a passive role. In a multinomial logistic regression analysis, younger age, female sex, lower morbidity, and higher education status were associated with preference of an active role [6].

In a US-American representative sample, 2383 adults without a history of cancer were confronted with a hypothetical cancer diagnosis and a moderate chance of survival. Asked for their treatment decision-making preferences, 8% preferred a passive role, 48% preferred a collaborative role, and 44% preferred an active role [7]. In this sample, respondents with higher education levels reported a higher preference for having a more active role in decision-making [7].

In several studies, decision preferences were also investigated in prostate cancer patients [8–16]. Preferences varied widely, but possible reasons for this observation were only investigated to a very limited extent.

The present study is intended to explore associations of the treatment decision-making preferences of prostate cancer patients with a wider range of variables. We investigated in the association between decision-making preferences and data collection mode (online vs. paper and pencil), participation in self-help groups, treatment status (before or after local treatment), radical prostatectomy (performed or planned vs. none), Gleason score, metastases, oncologic risk group, age, education, depression, anxiety, and quality of life.

## Methods

In four survey studies, we asked a total of 7169 prostate cancer patients about their decision-making preferences and various psychological variables relevant to the study objective. In the first two surveys, we interviewed 686 users of an online support group (OSG) [17] and 939 participants in face-to-face support groups [18]. In a third study, 920 prostate cancer patients were investigated in a follow-up survey of the HAROW study—a large health services research study on routine care in Germany [5, 19]. All of these studies had received positive ethics statements prior to initiation. A fourth group of 4624 participants anonymously answered questionnaires regarding an online decision aid for patients with newly diagnosed non-metastatic prostate cancer [20, 21]. For the present work, we used anonymized datasets derived from these studies. Therefore, no additional ethics statement was required.

## Control Preferences Scale

To determine the patients’ decision-making preferences, we applied a German version of the CPS [1]. Approximately 10 years ago, our working group had the original questionnaire translated and slightly adapted for questionnaire use. Since then, we have applied it successfully in several studies [22–25]. In this adapted version of the CPS, patients are asked to read five different statements about their desired type of participation in the decision-making process (Table 1). Afterwards, they choose the statement that they perceive to be closest to their preferred role in treatment decision-making [26]. They are asked to indicate only their first preference. Two statements describe more or less autonomous roles of the patient (active). The middle statement (I prefer that my doctor and I share responsibility for deciding which treatment is best) describes equal decision-making between the physician and patient (collaborative). Two additional statements describe the physician as having a more or less dominant role in decision-making (passive). Each answer can be interpreted within the range of autonomous to paternalistic on a 5-point ordinal scale (Table 1 and Appendix).

**Table 1:** Results of the Control Preferences Scale

		All	Data collection mode		*χ*^2^
		*N* = 7169	Online, *N* = 5530	Paper and pencil, *N* = 1639	*p*
Active	I prefer to make the final treatment selection	172 (2.4%)	113 (2.0%)	59 (3.6%)	< 0.001
	I prefer to make the final treatment selection after seriously considering my doctor’s opinion	2150 (30.0%)	1698 (30.7%)	452 (27.6%)	
Collaborative	I prefer that my doctor and I share responsibility for deciding which treatment is best	4456 (62.2%)	3484 (63.0%)	972 (59.3%)	
Passive	I prefer my doctor to make the final treatment decision, but only after my doctor has seriously considered my opinion	342 (4.8%)	227 (4.1%)	115 (7.0%)	
	I prefer to leave all treatment decisions to my doctor	49 (0.7%)	8 (0.1%)	41 (2.5%)	

## Clinical, Psychological, and Quality-of-Life Measures

In addition to the CPS score, we assessed the Gleason score (≤ 6 vs. 7 vs. ≥ 8), the D’Amico oncologic risk classification (low vs. intermediate vs. high), clinical evidence for distant metastases (yes vs. no), local treatment (before or after), and radical prostatectomy (performed or planned vs. none). We also distinguished between the following groups: patients who completed the survey online vs. those who completed a paper-and-pencil version of the questionnaire, self-help users vs. patients who did not report being in such a group, and patients before treatment vs. patients who had already undergone at least one treatment. We measured depression and anxiety for capacity-related reasons with the short form of the Patient Health Questionnaire (PHQ-2) and the Generalized Anxiety Disorder Scale (GAD-2). These screening questionnaires consist of two core depression items and two core anxiety items [27]. To assess general quality of life, we used two questions from the European Organisation for Research and Treatment of Cancer Quality of Life Questionnaire Core 30 (EORTC QLQ-C30), which is an overall quality-of-life scale. The values were transformed into scores ranging from 0 to 100, with higher scores representing better quality of life [28].

## Statistics

We used analysis of variance, the Kruskal-Wallis test, and the chi-square test to compare groups. We used a common slopes cumulative logit model for ordinal responses to identify the parameters associated with the preference for a more active role in treatment decision-making. A *p* < 0.05 was considered to indicate statistical significance. All calculations were performed with IBM SPSS Statistics 24 (Armonk, NY, USA) and SAS V9.4 (SAS Institute, Cary, NC, USA).

## Results

The mean age was 67.3 (SD: 7.8, median: 68, range: 39–94) years. Table 1 shows the control preferences for all patients and for the groups that completed the CPS online or with paper and pencil. Most patients (62.2%) preferred collaborative decision-making. A total of 2322 (32.4%) preferred an active role, and only 391 (5.5%) preferred a passive role. Patients using the paper-and-pencil survey preferred a passive role significantly more often than online users (9.5% vs. 4.2%, *p* < 0.001).

For the univariate analysis, we grouped all patients according to their control preferences. The characteristics of these groups are compared in Table 2. Significant differences between the control preference groups were found in most variables. Only depressiveness and anxiety did not differ significantly between the control preference groups.

**Table 2:** Comparison of medical data, age, quality of life, depression, and anxiety in the different control preference groups

	Active, *N* = 2322	Collaborative, *N* = 4456	Passive, *N* = 391	*p*
Age				
<51	77 (3.3%)	76 (1.7%)	6 (1.5%)	< 0.001^a^
51–60	467 (20.1%)	754 (17.0%)	59 (15.1%)	
61–70	1007 (43.4%)	1902 (42.8%)	148 (37.9%)	
> 70	769 (33.1%)	1716 (38.6%)	178 (45.5%)	
Education*				
Low	448 (23.4%)	1050 (29.1%)	76 (29.6%)	
Medium	510 (26.7%)	1078 (29.9%)	68 (26.5%)	< 0.001^c^
High	754 (39.4%)	1152 (31.9%)	85 (33.1%)	
Peer-to-peer support				
Self-help users	716 (30.8%)	867 (19.5%)	67 (17.1%)	< 0.001^c^
None	1606 (69.2%)	3589 (80.5%)	324 (82.9%)	
Treatment status				
Before treatment	1334 (57.5%)	3075 (69.0%)	215 (55.0%)	< 0.001^c^
After treatment	988 (42.5%)	1381 (31.0%)	176 (45.0%)	
Radical prostatectomy				
Performed/planned	1603 (69.0%)	3245 (72.8%)	317 (81.1%)	< 0.001^c^
None	719 (31.0%)	1211 (27.2%)	74 (18.9%)	
Gleason score				
≤ 6	859 (39.6%)	1468 (35.3%)	111 (31.1%)	
7	1029 (47.5%)	2036 (48.9%)	179 (50.1%)	< 0.001^k^
≥ 8	279 (12.9%)	656 (15.8%)	67 (18.8%)	
Metastatic disease				
M0/Mx	2101 (32.3%)	4059 (62.4%)	340 (5.2%)	0.10^c^
M1	92 (34.5%)	154 (57.7%)	21 (7.9%)	
Risk group				
Low	648 (29.5%)	1094 (25.9%)	86 (23.5%)	
Intermediate	866 (39.4%)	1734 (41.1%)	143 (39.1%)	0.003^k^
High	684 (31.1%)	1392 (33.0%)	137 (37.4%)	
EORTC score				
≤ 50	411 (17.9%)	843 (19.1%)	87 (22.5%)	
51–75	665 (28.9%)	1417 (32.1%)	132 (34.2%)	< 0.001^a^
≥ 76	1226 (53.3%)	2158 (48.8%)	167 (43.3%)	
PHQ-2	1.0 ± 1.3 0.5 (0–6)	1.0 ± 1.2 1 (0–6)	1.1 ± 1.4 1 (0–6)	0.14^a^
GAD-2	1.0 ± 1.3 1 (0–6)	1.0 ± 1.2 1 (0–6)	1.1 ± 1.4 1 (0–6)	0.056^a^

*Education was not assessed in the HAROW survey

^a^Analysis of variance

^c^Chi-square test

^k^Kruskal-Wallis test

The highest percentages of patients preferring an active role were found among the youngest age group (< 51 years: 48.4%), self-help users (43.4%), patients who had already undergone local treatment (38.8%), and patients with high education levels (37.9%).

Most patients preferring a collaborative style were patients who had low (66.5%) or medium (65.1%) education levels, had not yet undergone local treatment (66.5%), had a Gleason score ≥ 8 (65.5%), were not self-help users (65.0%), were in the oldest age group > 70 (64.4%), and had a moderate quality-of-life score of 51–75 (64.0%).

The highest percentages of patients preferring a passive role were observed among patients who were older than 70 years old (6.7%); had undergone prostatectomy (6.1%); had already undergone local treatment (6.9%); had low quality of life (≤ 50: 6.5%); and had higher oncologic risk, i.e. a Gleason score ≤ 8 (6.7%), metastatic disease (7.9%), and high oncologic risk (6.2%).

**Fig. 1:**
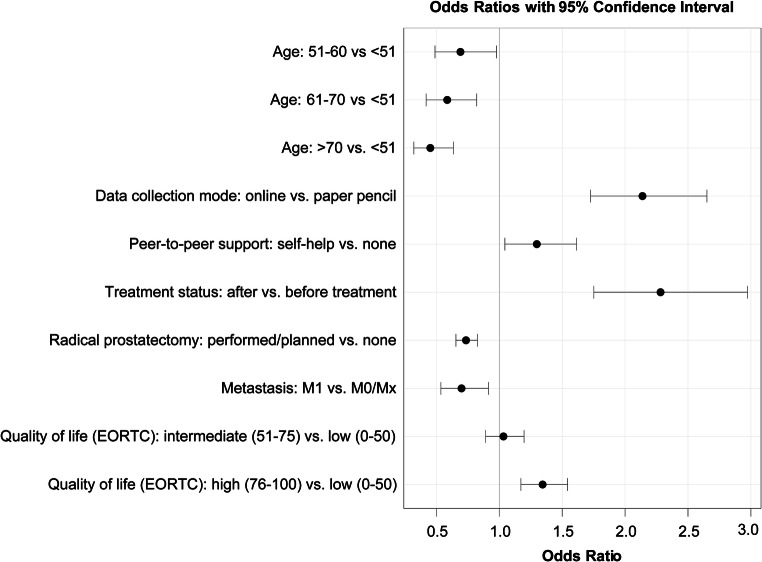
Significant variables of the common slopes cumulative logit model (*N* = 6703)

The results of the common slopes cumulative logit model for ordinal responses are presented in Fig. 1. In this model, age (*p* < 0.001), data collection mode (*p* < 0.001), peer-to-peer support (*p* = 0.018), treatment status (*p* < 0.001), radical prostatectomy (*p* < 0.001), metastatic disease (*p* = 0.001), and quality of life (*p* < 0.001) showed significant associations with the preferred decision-making role. Oncologic risk group, anxiety, and depression were not significant in this model.

## Discussion

Several previously undescribed variables related to treatment decision-making preferences in prostate cancer patients have been identified. We found significant associations between active decision-making preferences and young age, high education, online data collection mode, peer-to-peer support users, data collection before treatment, a not performed or planned radical prostatectomy, non-metastatic diseases, and high quality of life.

Regarding prostate cancer patients, the literature shows wide variation in preferences based on the CPS (Table 3). In an early investigation, the percentage of respondents with a preference for a passive role was the highest (58%) [8]. However, a few years later, in similar populations, only 8 to 10% wanted to play a passive role [9, 10]. In a recent investigation, 19% preferred a passive role [2]. Recently, 3348 prostate cancer survivors in Ireland were asked to describe their actual decision-making experience [11], and the percentages for each role were almost equally distributed (31% passive, 33% shared, and 36% active). Studies by our working group also showed large differences. For example, the percentage of users of an OSG who preferred active decision-making was almost twice as high (58%) as that of users of a face-to-face support group (33%) [17].

**Table 3:** Results of decision-making preferences based on the CPS in studies with prostate cancer patients

Authors and year	Population	Preferred decision-making role		
		Active (%)	Collaborative (%)	Passive (%)
Davison, Degner et al. 1995	57 Canadian prostate cancer patients	19	23	58
Davison, Gleave et al. 2002	80 Canadian prostate cancer patients	50	43	8
Davison, Parker et al. 2004	87 Canadian transrectal biopsy patients	43	47	10
Cuypers, Lamers et al. 2016	562 prostate cancer patients from the Netherlands	22	59	19
Drummond, Gavin et al. 2018	3348 prostate cancer survivors in Ireland	36	33	31
Ihrig, Maatouk et al. 2020	7169 German prostate cancer patients (pooled analysis)	32	62	6

The percentages of control preferences also varied widely between different countries. Compared with patients in most international studies, German prostate cancer patients very rarely (6%) preferred a passive role [16]. More than 30% of Spanish patients with various diseases [13] preferred a passive role. Additionally, in several international investigations of prostate cancer patients, the percentages of patients with passive decision-making preferences were high: 19% in the Netherlands [2], 31% in Ireland [11], and 11% in Japan [15]. We found two US studies reporting low percentages of patients with passive control preferences among primarily retired servicemen from the military with prostate cancer (3–4%) [12] and among newly diagnosed localized prostate cancer patients (6.5%) [14]. The differences described could be due to different language versions, effects of the respective zeitgeist, or national characteristics. However, these possible influences could not be examined in the present study.

### Timing

In our sample, the timing of the survey played an important role. Prostate cancer patients who were in an early stage, after diagnosis but before treatment started, more often favoured a collaborative role. The percentage who preferred a collaborative role was lower among patients who had already started or finished their treatment. They more often preferred an active or passive role. At a later stage of treatment, when they usually gained more experience, patients seemed to develop clearer preferences. Some patients might have felt more competent and therefore wanted to have more influence on the treatment decision. After patients had already made several decisions, the perceived importance of the remaining decisions might have decreased so that patients no longer needed their physicians’ help in the decision-making process. Another smaller proportion of patients tended to prefer a rather passive role after treatment. They might have either had positive experiences with physicians or simply become tired of making decisions and thus tended to leave decisions to their physicians.

### Age

The correlation between decision-making preferences and age was similar to the results of other investigations. Older patients more often prefer a passive role in treatment decision-making [2]. The same association was found in a study with 1529 men with newly diagnosed clinically localized prostate cancer [14]. A positive association between age and the preference for a passive role has also been observed for patients with other diseases [6].

### Self-help

To our knowledge, there are no results outside our working group showing a positive correlation between participation in self-help groups and an increased preference for an active role. However, the connection seems to be very understandable, as participation in self-help groups is particularly useful for prostate patients to gain information [29, 30] and would therefore be particularly interesting for patients who prefer an active role.

### Data Collection

More patients who completed the online version of the CPS than those who completed the paper-and-pencil version wished to play an active role in the decision-making process. Since a part of our sample had the choice of whether to complete the online or paper-and-pencil version, this choice might also have been linked to general use of the Internet. This would be in line with findings from 1945 older adults who had greater odds of preferring an active role in decision-making when using the Internet [31]. On the other hand, another part of our sample did not have the choice between the two options. Therefore, this connection cannot be conclusively determined based on our data.

### Prostatectomy

We found an association between the tendency of patients to opt for prostatectomy and a preference for a passive role in the decision-making process. This association could be explained by some interview statements provided by prostate cancer patients during external beam radiotherapy or their hospital stays shortly after radical prostatectomy. Patients who opted for surgery cited personal attitudes towards cancer and their desires to eradicate the cancer as quickly as possible as reasons for their treatment decisions and preferences [23]. A discussion of the treatment decision, which is a prerequisite for an informed active role, would not promote a quick solution. Other authors found that emotional distress increased the likelihood of undergoing surgery among men with localized prostate cancer [32]. However, in contrast to our results, another study of 3348 prostate cancer survivors showed that prostatectomy patients chose an active role more often than patients receiving other treatments [11].

### Clinical Characteristics

We found a significant correlation between having a metastatic disease and an increased preference for a passive role. In studies with non-prostate patients, Lechner et al. also reported that multimorbid patients preferred a passive role more often than people with no or only one chronic disease. Spanish patients with various diseases showed an increased preference for a passive role with increasing disease severity [13]. This connection could be due to the fact that patients do not want to trust their own knowledge in more complex health issues, but rather the expertise of physicians.

### Quality of Life, Depression, and Anxiety

Quality of life was highest among patients who preferred an active role and lowest among patients who preferred a passive role in decision-making. This association was also found in patients with myelodysplastic syndromes [33]. In a literature review of low-risk prostate cancer patients under active surveillance, 23 (55%) of 42 empirical assessments of decision-making preferences and quality of life (including general quality-of-life, depression, and anxiety measures) showed significant correlations between the two variables [34]. No significant association between treatment decision-making and global health–related quality of life was found in 3348 prostate cancer survivors [11]. We did not find significant correlations between depression and anxiety and control preferences. Also in the literature, we did not find any studies reporting a significant relationship between decision-making preference and the variables depression and anxiety.

### Limitations

There are several additional limitations in our study. As this is a cross-sectional study, no statements can be made about causality. The validity of the CPS, especially in different contexts, has been questioned [35]. There may be different interpretations, especially in the slight differentiation of active and passive roles. However, these subcategories are summarized in our calculations. Other instruments measuring decision-making preferences also show similar weaknesses [36]. Nevertheless, the CPS has been used in a large number of studies. In a systematic review from 2008, most studies on decision-making preferences used the CPS (Hubbard, Kidd et al. 2008). Additionally, 10 years later, a literature review on quality of life in low-risk prostate cancer patients found the CPS to be the second most common instrument used to assess this topic [34, 37]. Due to the different surveys on which our calculations are based, several problems occurred. The CPS was applied to patients in various situations, which could lead to different tendencies in the responses. In a recent study, the congruence between the roles actually experienced and the roles theoretically preferred in treatment decision-making was only 58% [11]. Since the parameters collected in the various surveys were somewhat different, several covariates could not be taken into account. Nevertheless, the large sample size enabled us to carry out an extensive evaluation with sufficient selection of relevant variables. Therefore, our evaluation provides a solid basis for describing relevant aspects of control preferences in prostate cancer patients.

## Conclusions

In particular, younger prostate cancer patients with higher quality of life completing an online survey want to play a more active role in treatment decision-making. Before treatment has started, patients tend to prefer collaborative decision-making. Few prostate cancer patients in Germany prefer a passive role. These patients are mostly older patients, patients with a metastatic disease, and patients who have opted for prostatectomy. Whether this finding reflects a generational effect or a tendency in different age groups and disease phases should be investigated. Further research is also needed to describe the causalities of these relationships. Moreover, we strongly believe that the CPS offers valuable information for personal counselling and should therefore be applied in clinical routine.

## Data Availability

Due to the nature of this research, participants of this study did not agree for their full data to be shared publicly, so complete data is not available. A reduced dataset of this study is available on request from the corresponding author, AI.

## Appendix

German CPS statements:

- Ich möchte selbst darüber entscheiden, welche medizinische Behandlung ich erhalte.
- Ich möchte letztendlich über meine medizinische Behandlung entscheiden, nachdem ich mich ernsthaft mit der Meinung meines Arztes auseinandergesetzt habe.
- Ich möchte, dass mein Arzt und ich die Verantwortung dafür teilen, welche Behandlung für mich am besten ist.
- Ich möchte, dass mein Arzt die endgültige Entscheidung über meine medizinische Behandlung trifft, meine Meinung dabei aber mit einbezieht.
- Ich möchte alle Entscheidungen, die meine medizinische Behandlung betreffen, meinem Arzt überlassen.

## Abbreviations

CPS: Control Preferences Scale
EORTC: European Organisation for Research and Treatment of Cancer
OR: odds ratio
HAROW: Hormone Therapy, Active Surveillance, Radiation, Operation, or Watchful Waiting
